# Measurement of Trunk Movement during Sit-to-Stand Motion Using Laser Range Finders: A Preliminary Study

**DOI:** 10.3390/s23042022

**Published:** 2023-02-10

**Authors:** Haruki Toda, Kiyohiro Omori, Katsuya Fukui, Takaaki Chin

**Affiliations:** Robot Rehabilitation Center, The Hyogo Institute of Assistive Technology, Kobe 651-2134, Hyogo, Japan

**Keywords:** joint angle, laser range finder, motion capture, sit-to-stand, trunk movement

## Abstract

The sit-to-stand (STS) motion evaluates physical functions in frail older adults. Mounting sensors or using a camera is necessary to measure trunk movement during STS motion. Therefore, we developed a simple measurement method by embedding laser range finders in the backrests and seats of chairs that can be used in daily life situations. The objective of this study was to validate the performance of the proposed measurement method in comparison with that of the optical motion capture (MoCap) system during STS motion. The STS motions of three healthy young adults were simultaneously measured under seven conditions using a chair with embedded sensors and the optical MoCap system. We evaluated the waveform similarity, absolute error, and relationship of the trunk joint angular excursions between these measurement methods. The experimental results indicated high waveform similarity in the trunk flexion phase regardless of STS conditions. Furthermore, a strong relationship was observed between the two measurement methods with respect to the angular excursion of the trunk flexion. Although the angular excursion of the trunk extension exhibited a large error, the developed chair with embedded sensors evaluated trunk flexion during the STS motion, which is a characteristic of frail older adults.

## 1. Introduction

The sit-to-stand (STS) motion is frequently performed in daily life, particularly before walking. As STS performance is associated with lower-limb muscle strength [1,2], this motion can be used to evaluate physical functions in older adults. The performance can be evaluated based on the time required for a certain number of repetitions, such as the five-times STS test, and the number of repetitions performed within a specified time, such as the 30-s chair-stand test. Compared with able-bodied adults, frail older adults require a significantly long time in the former test [3,4] and exhibit a low number of repetitions in the latter test [5,6]. Additionally, frail older adults have a low hip angular velocity and joint power when the trunk leans forward, resulting in a greater variability of the hip joint during STS motion [7]. Moreover, older adults have a smaller trunk flexion angle than young adults when rising from a seat [8]. Therefore, evaluating trunk movement during STS motion can serve as an effective screening method for frailty.

Generally, STS performance and joint kinematics are measured using specialized instruments such as stopwatches [3], inertial measurement units [9,10], and optical motion capture (MoCap) systems [11]. However, these instruments require evaluators and mounted sensors, in addition to restricting the measurement areas in laboratories. Consequently, measuring natural movement patterns by themselves is difficult for older adults in daily life situations without sensors attached to their bodies.

To address these problems, we developed a novel method for measuring STS motion using laser range finders wherein the distance between the sensor and target was measured. This sensor has previously been used for environmental detection [12]. In human movement analysis, a laser range finder can be used to measure spatiotemporal parameters, such as stride length, cadence, and foot trajectory, by measuring the distance from the chair to the leg during the timed up-and-go test [13,14]. Considering that the STS motion is performed without shifting the entire body, laser range finders embedded in the backrests and seats of chairs can theoretically calculate the trunk and thigh angles by combining them with the link model and measuring the distance from the chair during STS motion. In this study, we incorporated a simple two-link model with the trunk and thigh based on the knee joint in the sagittal plane. However, as the system was composed of inexpensive sensors and a simple model, the performance of the system had to be validated. As a preliminary step before producing a chair for measuring daily life scenarios, the effectiveness of the developed chair with embedded sensors was verified by assessing trunk movement during STS motion using a MoCap system. A comparative analysis of the two methods was performed to examine the validity of the trunk and thigh angles measured during STS motion.

## 2. Materials and Methods

### 2.1. Chair with Embedded Sensors

#### 2.1.1. Sensor Configuration

**Figure 1:**
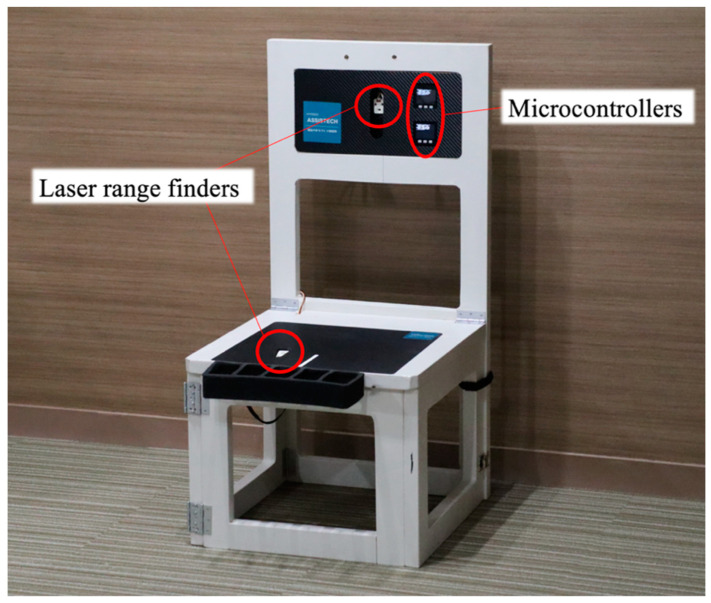
Overview of the developed chair with embedded sensors. The chair comprises two laser range finders to measure the distance between the chair and body. Microcontrollers used to control these sensors are embedded in the backrest.

Figure 1 depicts the developed chair with embedded laser range finders. The chair comprised two laser range finders (Time-of-Flight (ToF) Sensor Unit, VL53L0X, M5Stack, Shenzhen, China) to measure the distance between the seat and right thigh and that between the backrest and trunk. The distances were measured between the chair and the clothes using the ToF method. These sensors were connected to microcontrollers (M5Stack Basic V2.6, M5Stack, China) for sensor control. The microcontrollers were powered by mobile batteries embedded in the backrest. Data were stored using secure digital cards in each microcontroller. The sensor in the seat was embedded 80 mm to the right of the centerline. Additionally, a wired controller connected to each microcontroller was used to control the initiation and completion of the measurement. The height of the seat was set to 400 mm in accordance with a previous study that examined STS performance in older adults [6].

#### 2.1.2. Calculation of Segment Angles

**Figure 2:**
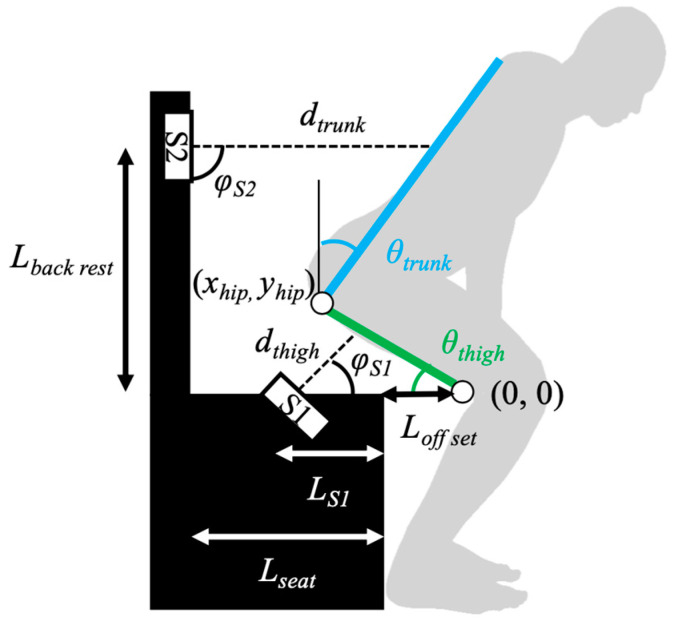
Link model assumed in this study. The two-link model for calculating the trunk angle (*θ_trunk_*) and thigh angle (*θ_thigh_*) originates from the knee joint.

Figure 2 depicts the two-link model used for calculating the trunk and thigh angles in this study. This model was incorporated to maintain a minimal configuration when calculating the trunk angle during STS motion. The distance data measured using the laser range finders were sampled at 30 Hz.

Initially, we calculated the thigh angle considering the distance from the seat to the thigh, as indicated in Equation (1).

(1) $$\theta_{thigh}=\mathrm{atan}\left(\frac{d_{thigh}\times \sin\left(\emptyset_{S1}\right)}{\left(L_{S1}+L_{off\;set}\right)-d_{thigh}\times \cos\left(\emptyset_{S1}\right)}\right)$$

where *d_thigh_* denotes the distance between the laser range finder embedded in the seat and thigh, *φ_S_*_1_ indicates the installation angle of the laser range finder embedded in the seat, *L_S_*_1_ represents the length between the laser range finder embedded in the seat and the edge of the seat, and *L_off set_* denotes the length between the edge of the seat and the knee joint center. In this study, *φ_S_*_1_, *L_S_*_1_, and *L_off set_* were set to 63.5°, 130 mm, and 50 mm, respectively.

Subsequently, we calculated the position corresponding to the hip joint using Equation (2), as follows:

(2) $$\left[\begin{array}{c} x_{hip}\\ y_{hip} \end{array}\right]=\left[\begin{array}{c} L_{thigh}\times \cos\left(\theta_{thigh}\right)\\ L_{thigh}\times \sin\left(\theta_{thigh}\right) \end{array}\right]$$

where *L_thigh_* denotes the thigh length. Our proposed method was not assumed when inputting users’ anthropometric data because it would be time-consuming to enter dimensional data each time before taking measurements in daily life. Thus, the model’s thigh length must be pre-set to one that can be adapted to different people. In this study, *L_thigh_* was set to 403 and 446 mm. Here, the former number was calculated from the fiftieth percentile of young Japanese men and women. The latter indicated the thigh length of the subjects participating in this study. which was calculated considering the heights of young men in the database of human body dimensions provided by the National Institute of Advanced Industrial Science and Technology (https://www.airc.aist.go.jp/dhrt/91-92/index.html, accessed on 1 November 2022).

Finally, we calculated the trunk angle based on the distance between the backrest and trunk using Equation (3).

(3) $$\theta_{trunk}=\mathrm{atan}\left(\frac{d_{trunk}\times \sin\left(\emptyset_{S2}\right)-\left(L_{seat}-x_{hip}\right)}{L_{S2-}d_{trunk}\times \cos\left(\emptyset_{S2}\right)-y_{hip}}\right)$$

where *d_trunk_* denotes the distance between the laser range finder embedded in the backrest and the trunk; *φ_S_*_2_ indicates the installation angle of the laser range finder embedded in the backrest; *L_seat_* represents the length of the seat; and *L_S_*_2_ denotes the length between the laser range finder embedded in the backrest and the seat. In this study, *φ_S_*_2_, *L_seat_*, and *L_S_*_2_ were set to 90°, 550 mm, and 465 mm, respectively.

### 2.2. Experiment

#### 2.2.1. Subjects

Three healthy young adults aged between 20 and 36 years participated in this study. The characteristics of the subjects can be summarized as follows:

- age: 21 years old, height: 1.86 m, weight: 73.4 kg;
- age: 22 years old, height: 1.80 m, weight: 65.8 kg;
- age: 36 years old, height: 1.81 m, weight: 78.0 kg.

The subjects exhibited no orthopedic disorder, neurological disorder, or pain in the lower limbs affecting their STS motion. This study was performed in accordance with the Declaration of Helsinki and approved by the Institutional Review Board of The Hyogo Institute of Assistive Technology (R2204).

#### 2.2.2. Data Collection

An optical MoCap system with eight infrared cameras (Vicon Vero, Vicon Motion Systems, Oxford, UK) was used to measure the marker trajectory at a sampling frequency of 100 Hz. Four reflective markers were attached to the C7 spinous process, sacrum, right greater trochanter, and right lateral femoral condyle.

**Figure 3:**
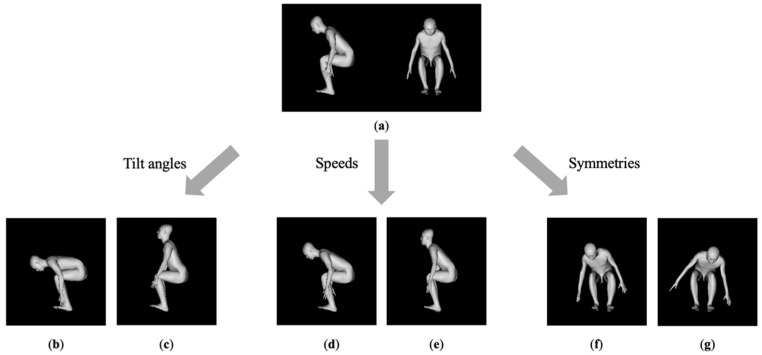
Experimental conditions analyzed in this study. (**a**) Normal; (**b**) Increase in the trunk anterior tilt; (**c**) Decrease in the trunk anterior tilt; (**d**) Slow speed; (**e**) Fast speed; (**f**) Right-side weight shift; (**g**) Left-side weight shift.

In addition to the normal comfortable motion, the subjects performed STS motions under seven conditions with varying trunk tilt angles, speeds, and symmetries (Figure 3). Under the trunk-tilt-angle condition, they performed the largest and smallest possible forward tilts. The speed condition measured the fastest and slowest possible movements. People with frailty, as well as healthy older adults, were considered under these tilt-angle and speed conditions [3,4]. Furthermore, the right and left weight-shift conditions were applied when considering people with asymmetrical lower limb functions, such as those with osteoarthritis and fracture. The subjects were instructed to relax their upper limbs and ensure that the limb did not contribute to the STS motion. These conditions were randomized, and five trials were performed for each condition.

#### 2.2.3. Data Analysis

The distances measured by the laser range finder and marker trajectories were digitally filtered using a fourth-order low-pass Butterworth filter with a cutoff frequency of 6 Hz. Spline interpolation was used to resample the marker trajectory to 30 Hz before performing the analysis.

We calculated the segment angles of the trunk and right thigh in the sagittal plane using marker trajectories to validate the angles measured by the developed chair with embedded laser range finders. The trunk angle was calculated as the angle between the line connecting the C7 spinous process and the sacrum (vertical line). The thigh angle was calculated as the angle between the right greater trochanter and right lateral femoral condyle (horizontal line). We defined the start of STS motion as a change in the trunk flexion angle of at least 1°.

**Figure 4:**
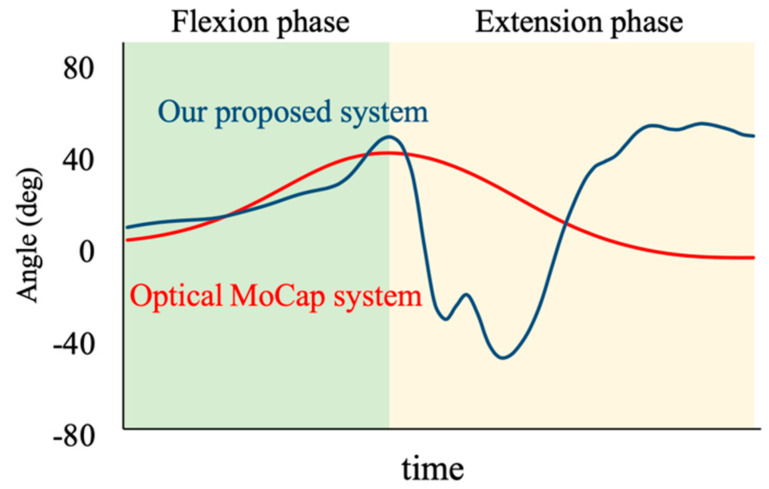
Typical time-series data of trunk flexion–extension angle obtained by our proposed system and optical motion capture (MoCap) system. Trunk flexion and extension excursions were calculated as the maximum displacement in the flexion and extension phase in each system.

Figure 4 shows a typical time series of trunk flexion angles obtained by our proposed system and optical MoCap system. Waveform similarities of the trunk and thigh angles were analyzed using cross-correlation between the calculations performed using the chair with embedded laser range finders and the optical MoCap system. In the case of the trunk angle, the cross-correlation coefficients were calculated by separating the flexion (between the timing at the start of STS motion and the timing at the maximum trunk flexion angle) and extension (between the timing at the maximum trunk flexion angle and the maximum extension angle) phases. Additionally, the absolute errors of trunk flexion and extension excursion in these phases during STS motion were also calculated using the proposed method and the optical MoCap system.

The values of five trials were averaged for the aforementioned parameters. To avoid floor and ceiling effects, the cross-correlation coefficients were normalized using Fisher’s r-to-z transformation. After averaging the values, a reverse z-transform was applied to convert them back to the correlation coefficients.

To evaluate the relationship between the angular excursions of trunk flexion and extension obtained from the proposed method and the optical MoCap system, Pearson’s correlation coefficients were calculated using all values from 35 trials (7 conditions × 5 trials) for each subject.

## 3. Results

### 3.1. Waveform Similarity

Table 1 summarizes the waveform similarity between the two measurement systems evaluated using cross-correlation coefficients. The thigh angles were over 0.800 in all conditions for all subjects.

Table 2 summarizes the cross-correlation coefficients calculated by separating the trunk angles according to the flexion and extension phases. The trunk angles in the flexion phase almost reached 0.900, whereas those in the extension phase exhibited large variations depending on the subjects and conditions. Minor effects of the thigh length setting were observed in all subjects.

### 3.2. Absolute Errors

Table 3 summarizes the differences between the two methods, namely the developed chair with embedded laser range finders and optical MoCap system, in terms of angular excursions in the flexion and extension phases. In subjects A and B, the absolute errors of flexion excursion in the normal condition were <3°. When altering the trunk anterior tilt, the absolute errors of flexion excursion increased in all subjects. The flexion angle of subject C exhibited a relatively large absolute error. Minor effects of the thigh length setting were observed in all subjects. By contrast, the extension angles exhibited large absolute errors in all conditions for all subjects.

### 3.3. Relationship of Joint Angular Excursions Calculated Using the Two Methods

**Figure 5:**
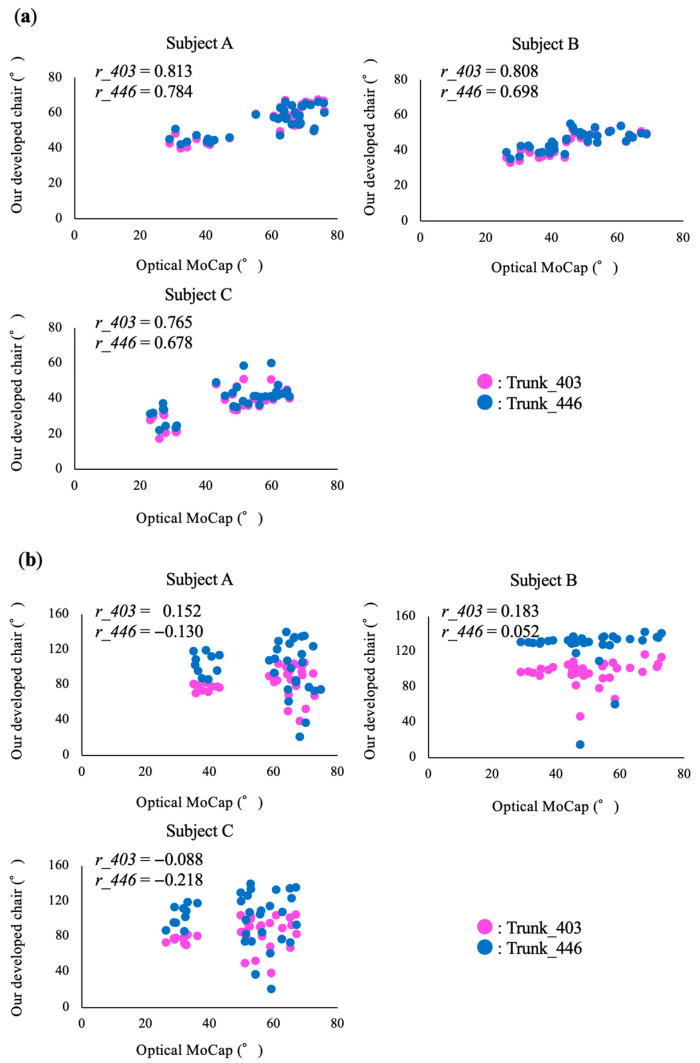
Scatter plots of angular excursion of (**a**) trunk flexion and (**b**) extension measured using the developed chair with embedded laser range finders and optical motion capture (MoCap) system. The chair with embedded laser range finders calculated trunk angles at two thigh-length settings, namely, 403 mm (Trunk_403) and 446 mm (Trunk_446). The correlation coefficients were calculated for each setting (*r_403* and *r_446*).

The mean values and standard deviation of the angular excursions of trunk flexion and extension obtained from the proposed method and the optical MoCap system in all trials are shown in Table 4. Figure 5 illustrates the relationship between the angular excursions of trunk flexion and extension calculated using the optical MoCap system and the chair with embedded laser range finders. The angular excursion of trunk flexion exhibited a moderate to strong correlation in all subjects regardless of the thigh length setting. Conversely, the correlation coefficients for the angular excursion of trunk extension were small.

## 4. Discussion

In this study, we examined the validity of the trunk and thigh angles during STS motion measured using laser range finders embedded in the backrest and seat of a chair. We confirmed that the waveform in the trunk flexion phase of the STS motion calculated using the proposed method was similar to that calculated by the optical MoCap system. By contrast, the waveforms in the extension phase exhibited less similarity with large absolute errors.

The trunk and thigh angles calculated based on the distance measured using laser range finders exhibited a great waveform similarity to the angles measured using the optical MoCap system regardless of the STS condition. Additionally, the correlation coefficients of trunk flexion excursion of these methods ranged from 0.6 to 0.8. These results indicate that the proposed measurement method can be used to evaluate trunk flexion during STS motion. Although healthy people produce STS motion using a momentum transfer strategy, the time required to execute the strategy is short and consistent as it is completed in one continuous motion [15]. To perform STS using this strategy, the horizontal momentum of the center of mass (CoM) is generated by the trunk flexion. The trunk flexion angle when rising from a seat of older adults tends to decrease in comparison with that observed in young adults [8]. The trunk flexion angle is negatively related to the knee extension moment, which is an indicator of knee mechanical stress in older adults [16]. In the future, we intend to evaluate the differences in trunk flexion angles between healthy and frail older adults using the chair with embedded laser range finders.

We applied two patterns based on thigh lengths of 403 and 446 mm, with the former representing the fiftieth percentile of young Japanese men and women and the latter indicating a value close to the actual thigh length of subjects. The difference in this parameter exhibited a minor impact on the obtained results. Consequently, we recommend a thigh length of 403 mm, which can be adapted to users of various heights in the case of young adults.

The absolute errors of trunk flexion excursion calculated using the proposed method and the optical MoCap system ranged from 4.5 to 41.4%. In subjects A and B, when STS conditions were increased and decreased in the trunk anterior tilt and right-side weight shift, respectively, the absolute errors of trunk flexion excursion were greater than 10°. As the extreme trunk flexion led to a discrepancy in the simple model by altering the horizontal CoM displacement [17], the absolute errors of trunk flexion excursion increased. Additionally, subject C exhibited relatively larger absolute errors in the trunk flexion excursion than subjects A and B, despite similar body shapes. This difference was attributed to inconsistent sitting positions and starting postures. Nevertheless, the proposed method was useful for evaluating trunk flexion during STS motion because the trunk flexion excursion measured by this method correlated with the value measured by the optical MoCap system.

The absolute errors of the extension phase were large, and the waveform similarities were small. Therefore, the proposed method failed to evaluate the trunk angle during this phase. These results were attributed to the use of the simple model based on the knee joint. The distance from the seat to the position of the knee axis was set as *L_off set_* = 50 mm. However, the position of the knee axis moved in the anteroposterior direction depending on the ankle joint motion. Particularly during the extension phase, the position of the knee axis moves in the posterior direction. The distance from the laser range finder embedded in the backrest was evaluated as the trunk was positioned posteriorly based on the assumed knee joint position. We speculated that this would increase the error of the trunk extension excursion. In this study, we applied the two-link model that required a minimal configuration involving two sensors to measure the trunk angle during STS motion for use in daily scenarios. If the purpose of measurement is limited to measuring the trunk flexion angle, the proposed chair with embedded laser range finders can be used clinically.

Nevertheless, certain limitations were noted in our analysis. First, only a few young adults participated in the study. Although the subjects performed various experimental conditions during STS motion, older adults may have a malalignment of the spine [18] in addition to abnormal motion patterns. Consequently, the calculation results in older adults are more likely to differ from those of young adults. Second, considering that frail older adults have low hip power to perform trunk flexion during STS motion [7], the effectiveness of the proposed method in evaluating their motion patterns is unclear. We intend to apply machine learning techniques to classify frailty status using STS parameters measured by the developed chair. Third, the measured distance data were the distances from chairs to clothes. Thus, errors are greater when measuring people wearing loose clothing or skirts. Finally, chairs used in daily life have many varying features, such as armrests and fabric cushioning. It is necessary to consider designs that fit into daily life while eliminating factors that affect standing.

## 5. Conclusions

This study proposes a simple method for measuring trunk movement during STS motion using laser range finders. The system uses an inexpensive sensor and a simple model for use in daily life scenarios. The effectiveness of the developed method was validated in the flexion phase during STS motion based on the results obtained from an optical MoCap system. In the future, we intend to evaluate the potential for using this system in determining trunk flexion in frail older adults.

## 6. Patents

The results of this study validate the recently published patent, “Motor function evaluation system, motor function evaluation method, and motor function evaluation program” JP Patent JP7134371B1 was filed on 31 January 2022 and issued on 9 September 2022.

## Figures and Tables

**Table 1:** Cross-correlation coefficients of the thigh angle during the sit-to-stand motion calculated using the chair with embedded laser range finders and the optical motion capture system.

Condition	Subject A	Subject B	Subject C
Normal	0.968	0.950	0.892
Increase in the trunk anterior tilt	0.961	0.902	0.902
Decrease in the trunk anterior tilt	0.965	0.927	0.832
Slow speed	0.967	0.848	0.863
Fast speed	0.871	0.912	0.845
Right-side weight shift	0.891	0.880	0.856
Left-side weight shift	0.959	0.901	0.910

**Table 2:** Cross-correlation coefficients of trunk angle separated by flexion and extension phase calculated using the chair with embedded laser range finders and the optical motion capture system.

Condition	Setting *	Subject A		Subject B		Subject C	
		Flexion	Extension	Flexion	Extension	Flexion	Extension
Normal	403	0.951	0.667	0.963	0.484	0.975	0.358
	446	0.979	0.713	0.943	0.357	0.961	0.320
Increase in the trunk anterior tilt	403	0.971	0.550	0.989	0.427	0.981	0.670
	446	0.993	0.598	0.979	0.337	0.971	0.685
Decrease in the trunk anterior tilt	403	0.844	0.367	0.978	0.251	0.964	0.134
	446	0.913	0.454	0.977	0.141	0.954	0.142
Slow speed	403	0.946	0.744	0.990	0.533	0.983	0.865
	446	0.968	0.820	0.979	0.614	0.978	0.935
Fast speed	403	0.870	0.156	0.963	0.160	0.944	0.094
	446	0.951	0.120	0.944	0.138	0.941	0.084
Right-side weight shift	403	0.987	0.899	0.981	0.560	0.980	0.989
	446	0.987	0.887	0.963	0.656	0.971	0.965
Left-side weight shift	403	0.848	0.703	0.979	0.598	0.979	0.447
	446	0.917	0.599	0.965	0.439	0.970	0.466

* The setting indicates the thigh length settings of the link model. 403 and 446 were the values calculated at thigh lengths of 403 and 446 mm, respectively.

**Table 3:** Absolute errors of joint angular excursions in the flexion and extension phases calculated using the chair with embedded laser range finders and the optical motion capture system.

Condition	Setting	Subject A		Subject B		Subject C	
		Flexion	Extension	Flexion	Extension	Flexion	Extension
Normal	403	2.2 (6.3)	23.2 (37.4)	0.6 (3.6)	51.8 (114.0)	12.1 (23.0)	31.3 (57.6)
	446	2.9 (7.1)	40.6 (65.2)	2.6 (6.8)	85.5 (188.2)	11.2 (21.1)	52.8 (96.7)
Increase in the trunk anterior tilt	403	6.3 (8.7)	21.3 (31.1)	16.8 (25.7)	37.3 (53.3)	19.3 (32.0)	31.1 (52.6)
	446	7.4 (10.2)	40.2 (58.7)	17.1 (26.2)	67.7 (96.7)	18.7 (30.9)	63.6 (108.4)
Decrease in the trunk anterior tilt	403	10.6 (33.5)	38.4 (101.3)	7.3 (25.1)	63.6 (194.9)	5.1 (20.3)	49.3 (204.9)
	446	13.1 (41.4)	55.3 (145.9)	9.8 (33.8)	97.6 (298.7)	8.0 (31.7)	82.8 (313.2)
Slow speed	403	2.9 (6.6)	20.8 (19.3)	6.9 (12.3)	34.6 (58.8)	15.0 (29.1)	10.8 (51.9)
	446	3.7 (7.1)	32.2 (26.4)	7.1 (12.5)	59.9 (101.7)	14.1 (28.4)	23.6 (86.9)
Fast speed	403	1.4 (4.8)	39.2 (102.7)	0.8 (8.4)	61.5 (148.3)	8.8 (30.4)	49.3 (153.0)
	446	2.1 (6.3)	75.8 (198.4)	1.1 (10.4)	91.4 (220.0)	5.5 (18.8)	82.9 (256.7)
Right-side weight shift	403	17.9 (25.1)	13.1 (19.3)	1.8 (4.5)	36.1 (68.1)	12.1 (20.8)	11.9 (61.4)
	446	16.4 (22.8)	13.7 (26.4)	0.2 (7.5)	57.3 (133.2)	8.4 (20.1)	6.7 (65.0)
Left-side weight shift	403	9.1 (14.0)	37.7 (57.7)	1.6 (4.9)	41.0 (84.8)	14.6 (25.6)	54.1 (97.8)
	446	9.0 (14.0)	68.8 (105.2)	2.9 (6.5)	76.5 (158.0)	13.6 (23.7)	82.8 (149.1)

Unit: degree (percentage of absolute error). The setting indicates the thigh-length setting of the link model. 403 and 446 denote the values calculated at thigh lengths of 403 and 446 mm, respectively.

**Table 4:** Joint angular excursions in the flexion and extension phases calculated using the chair with embedded laser range finders in two thigh length settings and the optical motion capture system in all trials.

Setting	Subject A		Subject B		Subject C	
	Flexion	Extension	Flexion	Extension	Flexion	Extension
Optical	58.6 (14.5)	58.4 (13.4)	46.8 (11.5)	50.1 (11.6)	47.9 (14.3)	49.8 (12.9)
403	54.8 (8.7)	82.4 (15.4)	44.3 (5.9)	96.7 (12.8)	36.9 (8.8)	82.1 (27.5)
446	45.7 (5.3)	101.2 (27.3)	45.7 (5.3)	126.7 (23.5)	38.8 (8.7)	107.7 (36.0)

Value: Mean (Standard Deviation). Unit: degree. Optical means the values of optical motion capture system. The setting indicates the thigh-length setting of the link model. 403 and 446 denote the values calculated at thigh lengths of 403 and 446 mm, respectively.

## Data Availability

Data sharing is not applicable.

## References

[1] Skelton D.A., Greig C.A., Davies J.M., Young A. (1994). Strength, power and related functional ability of healthy people aged 65–89 years. Age Ageing.

[2] Md J.F., Kiely D.K., Herman S., Leveille S.G., Mizer K., Frontera W.R., Fielding R.A. (2002). The relationship between leg power and physical performance in mobility-limited older people. J. Am. Geriatr. Soc.

[3] Schaubert K.L., Bohannon R.W. (2005). Reliability and validity of three strength measures obtained from community-dwelling elderly persons. J. Strength Cond. Res..

[4] McCarthy E.K., Horvat M.A., Holtsberg P.A., Wisenbaker J.M. (2004). Repeated chair stands as a measure of lower limb strength in sexagenarian women. J. Gerentol. A Biol. Sci. Med. Sci..

[5] Millor N., Lecumberri P., Gomez M., Martinez A., Martinikorena J., Rodriguez-Manas L., Garcia-Garcia F.J., Izquierdo M. (2017). Gait velocity and chair sit-stand-sit performance improves current frailty-status identification. IEEE Trans. Neural Syst. Rehabil. Eng..

[6] Jones C.J., Rikli R.E., Beam W.C. (1999). A 30-s chair-stand test as a measure of lower body strength in community-residing older adults. Res. Q. Exerc. Sport.

[7] Park C., Mishra R., Sharafkhaneh A., Bryant M.S., Nguyen C., Torres I., Naik A.D., Najafi B. (2021). Digital biomarker representing frailty phenotypes: The use of machine learning and sensor-based sit-to-stand test. Sensors.

[8] Gross M.M., Stevenson P.J., Charette S.L., Pyka G., Marcus R. (1998). Effect of muscle strength and movement speed on the biomechanics of rising from a chair in healthy elderly and young women. Gait Posture.

[9] Park C., Sharafkhaneh A., Bryant M.S., Nguyen C., Torres I., Najafi B. (2021). Toward remote assessment of physical frailty using sensor-based sit-to-stand test. J. Surg. Res..

[10] Regterschot G.R., Folkersma M., Zhang W., Baldus H., Stevens M., Zijlstra W. (2014). Sensitivity of sensor-based sit-to-stand peak power to the effects of training leg strength, leg power and balance in older adults. Gait Posture.

[11] Anan M., Hattori H., Tanimoto K., Wakimoto Y., Ibara T., Kito N., Shinkoda K. (2017). The coordination of joint movements during sit-to-stand motion in old adults: The uncontrolled manifold analysis. Phys. Ther. Res..

[12] Laković N., Brkić M., Batinić B., Bajić J., Rajs V., Kulundžić N. Application of low-cost VL53L0X ToF sensor for robot environment detection. Proceedings of the 2019 18th International Symposium INFOTEH-JAHORINA (INFOTEH).

[13] Adachi D., Nishiguchi S., Fukutani N., Hotta T., Tashiro Y., Morino S., Shirooka H., Nozaki Y., Hirata H., Yamaguchi M. (2017). Generating linear regression model to predict motor functions by use of laser range finder during TUG. J. Orthop. Sci..

[14] Fujita K., Iijima H., Eguchi R., Kuroiwa T., Sasaki T., Yokoyama Y., Koyama T., Nimura A., Kato R., Okawa A. (2020). Gait analysis of patients with distal radius fracture by using a novel laser Timed Up-and-Go system. Gait Posture.

[15] Hughes M.A., Weiner D.K., Schenkman M.L., Long R.M., Studenski S.A. (1994). Chair rise strategies in the elderly. Clin. Biomech..

[16] Nozawa R., Yamamoto S. (2012). The relationship of lower limb and trunk movements in sit-to-stand performed by the young and elderly. Rigakuryoho Kagaku.

[17] Mathiyakom W., McNitt-Gray J.L., Requejo P., Costa K. (2005). Modifying center of mass trajectory during sit-to-stand tasks redistributes the mechanical demand across the lower extremity joints. Clin. Biomech..

[18] Hirai T., Uehara M., Miyagi M., Takahashi S., Nakashima H. (2021). Current advances in spinal diseases of the elderly: Introduction to the special issue. J. Clin. Med..

